# Decoding the Nexus: Cellular and Molecular Mechanisms Linking Stroke and Neurotoxic Microenvironments in Brain Cancer Patients

**DOI:** 10.3390/biom14121507

**Published:** 2024-11-26

**Authors:** Spiro Menounos, Helen Shen, Shraddha Tipirneni, Sonu M. M. Bhaskar

**Affiliations:** 1 Global Health Neurology Lab, Sydney, NSW 2150, Australia; s.menounos1@gmail.com (S.M.); helen.shen@student.unsw.edu.au (H.S.); shraddha.tipirneni@student.unsw.edu.au (S.T.); 2 School of Clinical Medicine, Medicine & Health, University of New South Wales (UNSW), St George and Sutherland Clinical Campuses, Sydney, NSW 2150, Australia; 3 UNSW Medicine and Health, University of New South Wales (UNSW), South West Sydney Clinical Campuses, Sydney, NSW 2170, Australia; 4 NSW Brain Clot Bank, NSW Health Pathology, Sydney, NSW 2170, Australia; 5 Ingham Institute for Applied Medical Research, Clinical Sciences Stream, Liverpool, NSW 2170, Australia; 6 Department of Neurology & Neurophysiology, Liverpool Hospital and South West Sydney Local Health District, Liverpool, NSW 2150, Australia; 7 National Cerebral and Cardiovascular Center (NCVC), Department of Neurology, Division of Cerebrovascular Medicine and Neurology, Suita 564-8565, Osaka, Japan

**Keywords:** stroke, cancer, neurology, immunology, hypercoagulability, glioma, brain cancer

## Abstract

Stroke is an often underrecognized albeit significant complication in patients with brain cancer, arising from the intricate interplay between cancer biology and cerebrovascular health. This review delves into the multifactorial pathophysiological framework linking brain cancer to elevated stroke risk, with particular emphasis on the crucial role of the neurotoxic microenvironment (NTME). The NTME, characterized by oxidative stress, neuroinflammation, and blood–brain barrier (BBB) disruption, creates a milieu that promotes and sustains vascular and neuronal injury. Key pathogenic factors driving brain cancer-related stroke include cancer-related hypercoagulability, inflammatory and immunological mechanisms, and other tumor-associated processes, including direct tumor compression, infection-related sequelae, and treatment-related complications. Recent advances in genomic and proteomic profiling present promising opportunities for personalized medicine, enabling the identification of biomarkers—such as oncogenes and tumor suppressor genes—that predict stroke susceptibility and inform individualized therapeutic strategies. Targeting the NTME through antioxidants to alleviate oxidative stress, anti-inflammatory agents to mitigate neuroinflammation, and therapies aimed at reinforcing the BBB could pave the way for more effective stroke prevention and management strategies. This integrative approach holds the potential to reduce both the incidence and severity of stroke, ultimately improving clinical outcomes and quality of life for brain cancer patients. Further research and well-designed clinical trials are essential to validate these strategies and integrate them into routine clinical practice, thereby redefining the management of stroke risk in brain cancer patients.

## 1. Introduction

Stroke, albeit well-documented, remains an underappreciated complication in patients with brain cancer, reflecting a complex interplay between these two conditions. The convergence of stroke and brain cancer underpins a distinctive, multifactorial pathophysiological relationship [1]. This interplay is driven by three principal mechanisms: hypercoagulability associated with brain cancer [1], inflammatory and immunological dysregulation [2], and additional brain cancer tumor-specific processes. Emerging evidence suggests a unifying hypothesis implicating the formation and perpetuation of a neurotoxic micro-environment (NTME) as a central mechanism linking brain cancer and stroke.

Cancer-related hypercoagulability is prevalent in several malignancies, driven by the tumor-mediated expression of procoagulants and downregulation of natural anticoagulants [3]. Procoagulant cytokines, including interleukin (IL)-1, IL-6, and tumor necrosis factor (TNF)-a, promote the upregulation of tissue factor (TF) and cancer procoagulant (CP) expression [4], amplifying thrombotic risk. Pathological mechanisms such as venous thromboembolism (VTE) and paradoxical embolism via a patent foramen ovale (PFO) further contribute to stroke incidence in cancer patients [5,6]. Recent molecular studies have highlighted the role of specific brain cancer cell phenotypes in dysregulating the coagulation system through oncogene activation and tumor suppressor gene inactivation, directly modulating the expression of hemostasis-related genes [7,8,9].

Inflammatory and immunological pathways exacerbate this risk by fostering the NTME, characterized by oxidative stress, neuroinflammation, and blood–brain barrier (BBB) dysfunction, increasing susceptibility to severe strokes [10]. Additional tumor-related mechanisms include direct and indirect vascular occlusion, angioinvasion, cancer embolization [11], as well as infections and treatment-induced complications [12].

Given the significant morbidity and mortality associated with both stroke [13] and brain cancer [14], understanding these intersecting cellular and molecular mechanisms is imperative. This review highlights the NTME as a critical nexus in the pathophysiology of stroke in brain cancer, with particular emphasis on oxidative stress, neuroinflammation, and BBB disruption. Furthermore, we explore therapeutic strategies targeting the NTME, including interventions to modulate these processes and the potential of genomic and proteomic profiling to inform personalized medicine approaches.

## 2. Mechanisms of Brain Cancer-Related Stroke

**Figure 1:**
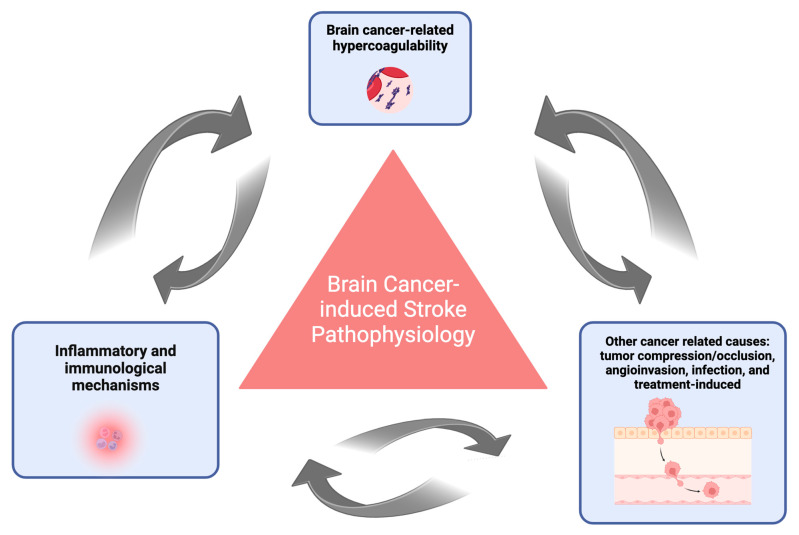
Pathophysiological framework of brain cancer-induced stroke. The pathogenesis of brain cancer-induced stroke is anchored by three interconnected pillars: cancer-related hypercoagulability, inflammatory and immunological dysregulation, and tumor-specific mechanisms. These processes include both direct and indirect vascular occlusion by tumors, angioinvasion, cancer-associated embolization, infections, and treatment-related complications. Figure 1 illustrates the dynamic interplay of these mechanisms, highlighting their synergistic role in stroke pathophysiology, while also contributing to cancer initiation and progression.

Cancer-related stroke is increasingly being recognized as a distinct stroke subtype, characterized by mechanisms that diverge significantly from those in non-cancer populations [1]. This unique pathogenesis encompasses three principal pathways (Figure 1) [15]: hypercoagulability associated with brain cancer, inflammatory and immunological dysregulation, and cancer-specific factors such as tumor compression, angioinvasion, infection, and treatment-related complications. These mechanisms do not function in isolation but interact synergistically, contributing to a spectrum of stroke presentations, including ischemic, hemorrhagic, or mixed types [16]. Elucidating these interconnected pathways is pivotal to developing targeted strategies to mitigate stroke risk and improve clinical outcomes for patients with brain cancer.

### 2.1. Brain Cancer-Related Hypercoagulability

**Figure 2:**
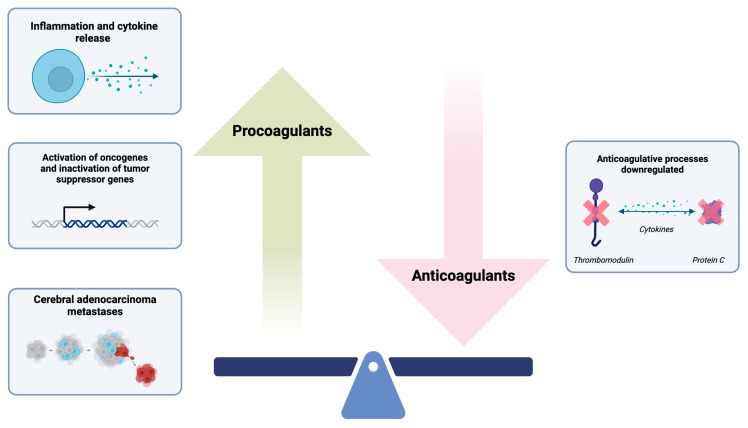
Mechanisms of brain cancer-related hypercoagulability. The tumor-induced upregulation of procoagulant factors, combined with the downregulation of natural anticoagulant mechanisms, facilitates thrombus formation. Key molecular pathways involved include cytokine-driven blood sludging, the upregulation of tissue factor and cancer procoagulant, mucin secretion, and the inhibition of protein C and thrombomodulin. These processes collectively contribute to a hypercoagulable state in brain cancer.

Coagulopathy associated with brain cancer represents a critical mechanism underlying the heightened risk of stroke in this patient population [12]. The hypercoagulable state in cancer arises from tumor-mediated upregulation of procoagulants and simultaneous suppression of natural anticoagulant pathway (Figure 2) [3]. Malignant cells drive the localized release of pro-inflammatory cytokines, including IL-1, IL-6, and TNF-a [4]. These cytokines alter vascular endothelial function, promoting red blood cell (RBC) aggregation along vessel walls (‘blood smudging’), which reduces luminal diameter and impairs blood flow.

In addition, these cytokines upregulate the expression of TF and CP in cancer cells, monocytes, and endothelial cells, amplifying inflammatory responses and the coagulation cascade [17]. TF catalyzes the activation of factors IX and X, promoting thrombin generation and facilitating thrombus formation, ultimately leading to stroke [18]. Elevated TF levels, frequently identified in atherosclerotic plaques, have been implicated in atherothrombosis by destabilizing plaques, further linking TF to cancer-associated hypercoagulability [19]. CP, a cysteine protease expressed almost exclusively in malignant cells, directly activates factor X independently of factor VII, further enhancing thromboembolic processes [20]. In addition, the tumor-driven cytokines IL-1, IL-6, and TNF-a suppress natural anticoagulants such as protein C and thrombomodulin, further exacerbating the coagulopathic state in brain cancer [21,22]. This intricate interplay between pro-inflammatory, procoagulant, and tumor-specific mechanisms underscores the central role of hypercoagulability in brain cancer-induced stroke.

Metastatic cerebral adenocarcinoma exacerbates a hyperviscous and hypercoagulable state through mucin production, a high-molecular-weight-molecule that acts as a potent procoagulant [23,24]. Mucin interacts with endothelial cells, platelets, and lymphocyte cell adhesion molecules, inducing the formation of platelet-rich microthrombi that deposit in vessels and cause ischemic stroke [24]. There is growing evidence of neutrophil extracellular trap (NET) burden in cancer-associated thrombosis [25]. Activated neutrophils, as part of the innate immune response, release decondensed chromatin to form NETs. This action promotes coagulation factor and platelet activation, as well as facilitates downstream thrombosis. By creating a scaffold for RBCs, platelets, and fibrinogen adhesion, both intrinsic and extrinsic coagulation pathways are activated. This action was further demonstrated by Thålin and colleagues, who found increased NET formation levels in patients with cancer-associated ischemic stroke compared to controls, strongly supporting a cancer-induced systemic pro-thrombotic state [25]. Disseminated intravascular coagulation (DIC) disrupts the balance between thrombolysis and thrombi formation. This disruption leads to either thrombotic vascular occlusion due to the overexpression of the coagulation cascade or diffuse hemorrhage secondary to the coagulation factor and platelet depletion [26]. Albeit a rare mechanism of coagulopathy in patients with primary brain tumors such as glioblastoma, patients with adenocarcinoma have been reported to be at a very high risk of DIC [27,28]. Therefore, patients with metastatic cerebral adenocarcinoma may be at an increased risk of complex thrombo–hemorrhagic processes, including manifestations of thrombotic stroke and intracerebral hemorrhage [29].

Ischemic stroke via a patent foramen ovale and paradoxical embolization from deep venous thrombosis (DVT) is another crucial mechanism involved in brain cancer-related hypercoagulability. Patients with primary or metastatic brain tumors are at an increased risk of venous thromboembolism (VTE), compared to other cancer types [5]. Clinically, the incidence of VTE ranges from 20% in metastatic brain disease to 30% in patients with high-grade glioma [30,31]. A microscopic assessment of surgically resected human glioblastoma multiforme (GBM) tissue revealed thrombotic vascular occlusion in 94% of tumors [32]. A prospective study of 184 ischemic stroke patients revealed that right-to-left shunts were significantly more common in cancer patients than in those without malignancy, at 55% to 15%, respectively [6]. This finding underscores the importance of routine assessments to investigate the presence of a PFO in brain cancer patients who develop DVT and/or ischemic stroke. Non-bacterial thrombotic endocarditis (NBTE), characterized as non-infectious, sterile valvular vegetations with negative blood cultures, is most commonly associated with underlying malignancy [33]. The pathophysiology involves cancer-related hypercoagulability, which predisposes patients to the formation of sterile platelet–thrombin vegetations on cardiac valves, with cerebral infarction as a late complication. NBTE is most frequently linked to adenocarcinomas and has also been reported in cases of primary malignant gliomas and metastatic cerebral lesions [34].

Cancer-related coagulopathy and Trousseau’s syndrome have traditionally been considered as “unspecific” consequences, representing the first physiological response to cancer-induced tissue injury. However, recent molecular studies suggest that specific cancer cell phenotypes may play an important role and affect the coagulation system. The activation of oncogenes (including RAS or EGFR) and the inactivation of tumor suppressor genes (including p53) directly affect the hemostasis-controlling gene expression [7,8,9]. Notably, different brain tumor subtypes, such as GBM and medulloblastoma, have demonstrated varying coagulome profiles. This finding suggests a potential correlation between tumorigenesis and specific procoagulant phenotypes expressed by malignant cells [35]. When comparing primary brain tumor subtypes, GBM remains the most deadly and is associated with thrombotic complications, extensive angiogenesis, and up-regulated TF. An analysis of classic GBM revealed that TF upregulation is driven by the expression of the oncogenic epidermal growth factor receptor (EGFR) and its mutant EGFR variant III (EGFRvIII) [36,37]. TF expression and GBM cells’ procoagulant activity were triggered under hypoxic conditions or together with EGFRvIII. This finding demonstrates that the expression of a particular procoagulant phenotype in tumor cells is driven by the activation of specific oncogenic pathways rather than isolated mutations [8,36]. In contrast, the proneural subtype of GBM, which frequently harbors an isocitrate dehydrogenase one mutation, is associated with decreased TF expression [38]. In addition, the GBM mesenchymal subtype exhibits TF downregulation, a pronounced inflammatory status, and elevated levels of plasminogen activator inhibitors 1, thrombomodulin, and endothelial protein C receptor [39,40]. These observations suggest that different brain cancer cell phenotypes may induce coagulopathies through various oncogene activations and tumor suppressor gene inactivations that directly influence hemostasis-controlling genes. Beyond hypercoagulability, key processes in brain cancer-related stroke development include the establishment of the NTME through inflammatory and immunological pathways.

### 2.2. The Neurotoxic Microenvironment

The establishment of an NTME is driven by oxidative stress and neuroinflammation, both of which contribute to cellular damage [41,42]. These processes disrupt the BBB, exacerbating the immunosuppressive milieu of the NTME [43]. Tumor cells further contribute to BBB dysfunction through direct invasion and the promotion of a pro-inflammatory state. The NTME is sustained by local tumor-related factors and systemic inflammatory processes, which synergistically support cancer initiation, progression, and malignant cell survival [44]. This inflammatory and oxidative landscape also amplifies stroke risk in brain cancer patients by fostering vascular and neuronal injury. Key contributors include systemic inflammation [45,46], cytokine release, and oxidative stress, which together promote a prothrombotic state [47,48].

Studies show that patients with brain cancer are predisposed to more severe strokes, often involving multiple vascular territories, and face poorer clinical outcomes (Table 1) [4,49,50,51,52]. Retrospective single-center studies have consistently demonstrated that ischemic stroke patients with cancer tend to be younger, present with more severe strokes on admission, and more frequently experience cryptogenic strokes or infarcts involving multiple vascular territories. Functional outcomes are notably worse compared to ischemic stroke patients without active cancer [49,50,51]. A prospective case-controlled study of 140 cancer patients matched with controls also demonstrated a significantly higher prevalence of cryptogenic strokes and multifocal infarctions in cancer patients [4].

Although some studies exclude patients with primary brain cancer or metastatic disease due to varying stroke mechanisms, those that include metastatic disease while excluding primary brain cancer report a particularly high stroke risk in metastatic cases, with a strong association with increased six-month mortality [4]. This finding suggests that current demographic data on cancer-related strokes, especially those involving brain cancer, may be underrepresented in the literature. A recent nationwide analysis of over 1.1 million hospitalizations in the United States (2015–2017) further underscores these findings. Cancer patients, including those with primary and metastatic brain cancer, exhibited increased in-hospital mortality, prolonged hospital stays, and reduced rates of routine discharge compared with non-cancer stroke patients [52]. These data highlight the unique and severe impact of the NTME in brain cancer-associated stroke.

#### 2.2.1. Inflammatory and Immunological Processes

Oxidative stress plays a central role in the cellular dysfunction underlying cancer initiation and progression. As cancer cells proliferate, they demand increasing amounts of ATP, leading to the generation and accumulation of reactive oxygen species (ROS). These molecules contribute to tumorigenesis through various mechanisms, including inflammation, immune evasion, angiogenesis, and DNA damage. Such processes induce the hyperexpression of proto-oncogenes and the inactivation of tumor suppressor genes [58]. ROS inhibit key tumor suppressive pathways, including the p53-mediated response, and impair the expression of endogenous antioxidant enzymes such as superoxide dismutase two and catalase [59]. Moreover, ROS activate the mitogen-activated protein kinase (MAPK) pathway, which drives the production of pro-inflammatory cytokines like IL-1, IL-6, TNF-α, and TGF-β, leading to nuclear factor-kappa B (NF-κB) activation and tumor proliferation [60]. Through these pathways, oxidative stress promotes chronic inflammation and cancer-associated hypercoagulability. Additionally, oxidative stress upregulates immune checkpoint molecules, such as programmed death-1 (PD-1), its ligand PD-L1, and cytotoxic T-lymphocyte-associated protein 4 (CTLA-4), which, while critical for preventing autoimmunity, are co-opted by cancer cells to evade anti-tumor immune responses. This dampening of T-cell activity facilitates tumor growth and impairs the immune system’s capacity to respond to other threats, such as stroke [61,62].

The brain is particularly susceptible to ROS and reactive nitrogen species (RNS) due to its high oxygen demand, abundance of peroxidizable lipids, and low antioxidant capacity in neurons [63]. Even minor reductions in oxygen and nitrogen levels, whether during normal brain function or in pathological states, trigger ROS and RNS generation from microglia and astrocytes [64]. In the context of brain ischemia and reperfusion, ROS and RNS play a pivotal role in oxidative-mediated tissue injury, initiating apoptosis, necrosis, and autophagy. These reactive species contribute to ischemic damage through lipid peroxidation, macromolecule crosslinking (including DNA and RNA), endothelial disruption, increased BBB permeability, neuroinflammation, edema, and cell death [65].

The synergistic effects of oxidative stress and neuroinflammation are crucial in establishing the NTME and are integral to the ischemic cascade. Nerve membranes and blood vessels, rich in polyunsaturated fatty acids, are primary targets for free radical-induced damage, leading to lipid peroxidation, protein denaturation, and cellular disruption. These processes compromise barrier functions, ion transport, and cell membrane integrity, exacerbating neuronal injury [65,66]. ROS and RNS, such as nitric oxide, promote cytotoxicity by inhibiting ATP-producing enzymes and activating pro-inflammatory enzymes like cyclooxygenase-2 (COX-2) [67]. These species also activate inflammatory cells, perpetuating a vicious cycle of ROS-mediated inflammation, with NF-κB further stimulating microglia, macrophages, and neutrophils [68].

In the context of cerebral ischemia and reperfusion, neuro-apoptosis is driven by the aberrant activation of intrinsic and extrinsic apoptotic pathways [69]. ROS such as hydrogen peroxide (H_2_O_2_) activate nuclear transcription factors like p53 and NF-κB, which in turn upregulate proteins that inhibit survival or promote cell death. ROS contribute to DNA damage, including single-strand breaks, which activate DNA-dependent kinases and ATM proteins, stabilizing and activating p53, a key regulator of neuronal survival and death following stroke [70,71]. Moreover, oxidative stress and ROS generation in conditions such as cellular stress, hypoxia, and ischemia are significant stimuli for autophagy, with H_2_O_2_ inhibiting key autophagic proteins such as Bcl-2 and Beclin-1, promoting autophagosome maturation and cellular death [72,73,74]. Thus, ROS and neuroinflammation not only mediate the development of the NTME but likely contribute to a preconditioning or predisposition for stroke in the context of brain cancer. While these inflammatory and immune processes directly induce neurotoxicity, they also lead to indirect damage through the disruption of the BBB.

#### 2.2.2. Blood–Brain Barrier Dysfunction

Primary brain tumors and metastatic cerebral disease contribute significantly to the NTME by disrupting the integrity of the BBB through a range of structural and functional alterations [43]. Oxidative stress-induced BBB breakdown occurs in two phases: the first involves direct endothelial dysfunction and BBB disruption, while the second is characterized by secondary damage driven by neuroinflammation, cell necrosis, and the activation of matrix metalloproteinases (MMPs) [75,76]. As brain tumors progress, they release the vascular endothelial growth factor (VEGF), a key mediator of angiogenesis that induces the formation of abnormal, permeable blood vessels, further reinforcing the NTME [77]. These new vessels lack the tightly regulated endothelial junctions typical of the intact BBB, leading to increased permeability. As a result, this disruption allows the entry of pro-inflammatory cells, toxins, and other harmful substances into the brain parenchyma, setting the stage for cerebrovascular damage and stroke [43]. Tumor-induced BBB dysfunction not only increases stroke risk but also impedes the delivery of therapeutic agents to the brain, complicating treatment approaches [78].

**Figure 3:**
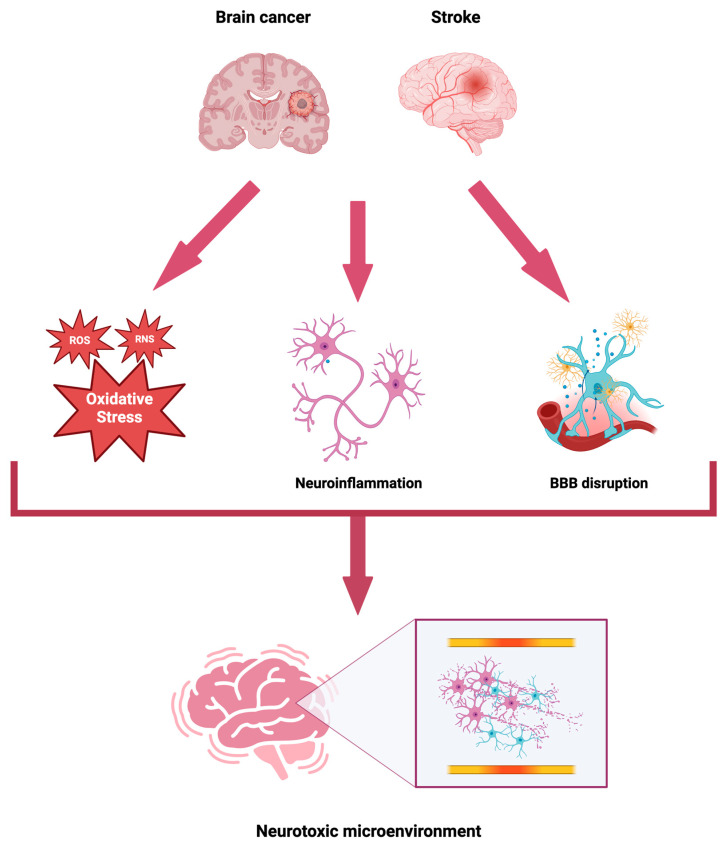
Inflammatory and immunological pathways in brain cancer-related stroke. This figure illustrates the inflammatory and immunological pathways that contribute to the establishment of a neurotoxic microenvironment (NTME) in brain cancer, thereby increasing stroke risk. It highlights the roles of oxidative stress, neuroinflammation, and blood–brain barrier (BBB) dysfunction in promoting cerebrovascular damage. The diagram also emphasizes how these interconnected processes exacerbate stroke severity and identifies potential molecular targets for therapeutic intervention. Abbreviations: ROS—Reactive oxygen species; RNS—Reactive nitrogen species; BBB—Blood-brain barrier.

Malignant brain edema, a leading cause of early mortality following ischemic stroke [79], arises from BBB rupture, resulting in tissue constriction and edema. This process reduces cerebral perfusion and increases regional tissue hydrostatic pressure, further aggravating cerebrovascular injury [80]. ROS and RNS are significant contributors to the development of both cytotoxic and vasogenic edema. Cytotoxic edema, a major determinant of stroke severity and final infarct size, is primarily driven by the depth and duration of ischemia. ROS-induced membrane phospholipid peroxidation and protein modifications of ion transporters contribute significantly to the pathogenesis of cytotoxic edema [81,82]. Moreover, increased VEGF expression, stimulated by both ROS and RNS, enhances vascular permeability, exacerbating vasogenic edema [83]. Together, these mechanisms suggest that the NTME present in brain cancer promotes cellular and molecular pathways that predispose the brain to infarction, potentially exacerbating cerebral damage (Figure 3). Beyond inflammatory and immunological mechanisms that sustain the NTME, brain cancer can also induce stroke through both direct and indirect processes, including tumor occlusion, angioinvasion, infections, treatment-related effects, and cancer embolization.

The phenotype of brain tumors plays a crucial role in BBB integrity. In both primary and metastatic brain tumors, BBB structure is compromised, facilitating increased permeability and the influx of neurotoxic substances. This disruption undermines the protective function of the BBB and contributes to the formation of the blood–tumor barrier, a highly heterogeneous structure [84]. Previous studies have shown that BBB disruptions can have systemic effects, elevating the risk of stroke [85]. The anatomical localization of tumors may also contribute to BBB vulnerability and the associated stroke risk. We postulate that disruption of the BBB may signal a transition from localized disease to a more systemic manifestation, as tumor growth eventually compromises the barrier, leading to systemic crossover. Thus, BBB disruptions may also be linked to systemic inflammation [45], further increasing the risk of stroke.

### 2.3. Direct Brain Tumor Compression

Direct brain tumor compression is relatively rare and can manifest in various forms. Ischemic stroke is secondary to the direct tumor compression of large vessels and is primarily seen in GBM, particularly affecting the middle cerebral artery (MCA) [86]. Other mechanisms of direct tumor compression include the leptomeningeal infiltration of arterial and venous sinuses and/or intratumoral hemorrhage [16,24]. Leptomeningeal carcinomatosis, also known as neoplastic meningitis, involves a malignant spread into the cerebrospinal fluid and is most commonly caused by malignant melanoma, lung and breast cancer [87]. The mechanism by which cerebral infarction occurs is due to perivascular space tumor growth, causing spasm or vessel thrombosis [88]. Sinovenous thrombosis most commonly occurs in the pediatric population. The underlying mechanism causing cerebral ischemia involves occlusion of the cerebral venous system, which obstructs venous outflow, leading to intracranial hypertension [89]. Intratumoral hemorrhage is the most common cause of intracranial hemorrhage in cancer patients [90]. This hemorrhagic mechanism is multifaceted and involves the rupture of newly formed fragile intra-tumoral vessels by angiogenesis and the direct angioinvasion of pre-existing vasculature structures [91]. Given the highly vasculitic nature, GBM is the most common primary brain tumor associated with intratumoral hemorrhage [92]. Indirect brain tumor compression of intracranial vascular structures can occur secondary to edema associated with primary tumor growth and/or high metastatic disease burden [93]. The compression of vascular structures, including venous drainage pathways, increases capillary bed pressure and can result in cerebral ischemia and infarction [94]. Another rare cause of cancer-related stroke includes embolic phenomena when primary cancer metastasizes to the heart, such as the esophagus, breast, lung, or melanoma [95].

### 2.4. Infection-Related Processes

Substantially elevated risks of infection due to immunocompromise not only increase the risk of infection sequelae but also significantly increase the risk of both hemorrhagic and ischemic stroke [96,97]. Organisms most commonly associated with a greater risk of stroke include Mycoplasma pneumonia, Chlamydia pneumonia, Helicobacter pylori, Hemophilus influenza, Epstein–Barr virus, cytomegalovirus, Herpes simplex virus (HSV)-1 and 2 [98]. The systemic inflammatory response, as a consequence of infection, can damage the vascular endothelium, predisposing patients to intracranial hemorrhage [99]. Mycotic aneurysms are formed from bacteria or septic emboli-mediated arterial wall degradation. These aneurysms occur at the distal portions of the MCA and can be numerous, with their rupture associated with a high mortality rate [100]. Other causes of hemorrhage include petechial cerebral hemorrhages mediated by HSV-1 meningoencephalitis [101].

From an ischemic perspective, syphilis can lead to ischemic stroke through progressive luminal stenosis and, in turn, thrombosis. This mechanism involves the development of obliterating endarteritis, characterized by vascular intima fibroblast proliferation, media thinning and adventitial inflammatory processes, and eventual fibrosis [102]. Notably, strokes accounted for approximately 10% of neurosyphilis presentations from a single-center analysis of 241 patients [103]. Among infective causes of ischemic stroke, septic embolization from infective endocarditis is common. This frequency was demonstrated in a prospective cohort study across 25 countries, including 2781 infective endocarditis adult inpatients, where stroke occurred in 17% [104]. Interestingly, infective endocarditis complicated by intracranial hemorrhage was most commonly identified with Streptococcus viridans, β-hemolytic streptococci, and Staphylococcus aureus [105].

Several viruses have also been implicated in a greater risk of ischemic and hemorrhagic stroke. Varicella zoster virus (VZV)-mediated vasculopathy associated with either acute infection (chickenpox) or reactivation can lead to stroke [106]. These VZV-related strokes typically affect deep brain structures such as the internal capsule and basal ganglia, as well as the cerebral cortex [107]. The Herpes zoster virus involving the trigeminal ganglia significantly increases the risk of stroke, also thought to be secondary to vasculopathy [108]. Other viruses implicated in stroke include enterovirus, West Nile virus, and parvovirus B19 [109,110]. In addition, several fungi have also been involved in stroke, such as molds, yeasts, and dimorphic fungi. In fungal meningitis, commonly caused by yeasts such as candida or cryptococcus spp., the underlying mechanism can involve venous outflow obstruction, significant vessel vasculopathy within the subarachnoid space, endarteritis, and abscess formation [111,112].

### 2.5. Treatment-Induced Complications

Therapies for brain cancer are associated with significant systemic side effects that increase the risk of stroke [113]. Evidence from studies of traditional cancer therapies, such as chemotherapy and radiotherapy indicates an elevated stroke risk [114], although the exact pathological mechanisms remains poorly understood [115,116,117]. Two primary mechanisms have been proposed to explain radiotherapy-related stroke predisposition in cancer patients: accelerated atherosclerosis driven by chronic inflammatory processes, and non-atherosclerotic cerebral vasculopathy [118]. A recent meta-analysis of over 55,000 cancer patients, including those with brain cancer, revealed that radiotherapy was associated with a twofold increase in the risk of subsequent stroke compared with non-irradiated controls [117]. In addition to radiotherapy, chemotherapy agents, such as cisplatin-based regimens commonly used in brain cancer treatment, are strongly implicated in thrombotic risks [15]. The underlying mechanisms include endothelial toxicity, the dysregulation of coagulation and hemostasis factors, and increased thrombin production facilitated by extracellular vesicles (EVs) and cell-free DNA by cancer cells [119,120].

EVs contain diverse bioactive molecules, including long non-coding RNAs, mRNAs, microRNAs, oncoproteins, oncopeptides, DNA fragments, and lipids, which modulate the tumor microenvironment [121]. These vesicles can propagate pro-thrombotic signals, contributing to systemic coagulation abnormalities. Importantly, thrombotic events linked to chemotherapy display distinct pathological patterns, encompassing both cerebral stroke and VTE, in contrast to the localized vascular damage predominantly seen with radiotherapy [122].

Angiogenesis inhibitors, such as bevacizumab, a VEGF inhibitor, are used in the treatment of highly vascularized brain tumors, including GBM [123]. A meta-analysis of 12,917 cancer patients treated with bevacizumab revealed a more-than-threefold increased risk of both CNS ischemic and hemorrhagic events compared to patients not receiving the agent [124]. The mechanism behind this elevated stroke risk is multifactorial. Angiogenesis inhibitors can induce endothelial damage, promoting fibrin formation, which subsequently increases the risk of ischemic stroke. Anti-angiogenic-induced hypertensive states exacerbate shear stress on atherosclerotic plaques, enhancing thrombotic activity. These inhibitors also create environments rich in lipoproteins, free fatty acids, and hyperglycemia, which further amplify the risk of atherothrombotic events [125].

The novel immune checkpoint inhibitors (ICIs) have revolutionized oncology, showing unprecedented success in treating primary brain cancers and brain metastases, although their efficacy in these contexts remains debated [126]. Targeted immune checkpoints include cytotoxic T lymphocyte antigen 4 (CTLA-4) and programmed cell death protein 1 (PD-1), with ipilimumab (anti-CTLA-4) and pembrolizumab (anti-PD-1) being the most prominent agents [126]. While these therapies have shown promising outcomes in cancer treatment, they have been associated with an increased risk of stroke. A recent single center, matched cohort study found a threefold higher incidence of cardiovascular events, including ischemic stroke, in patients who commenced ICI treatment compared to controls [127]. Similarly, Chiang et al. reported that cancer patients receiving ICIs had a higher likelihood of ischemic stroke compared to those not treated with ICIs [128]. The pathophysiological mechanisms behind this heightened stroke risk are not fully understood but are believed to involve inflammatory-induced vasculitis and endothelial damage, predisposed to stroke [127,129]. Animal models have supported this hypothesis, with PD-1 inhibition linked to increased atherosclerotic plaque formation through increased vascular adhesion and the infiltration of CD4-/CD8-positive T-cells [130,131].

Chimeric antigen receptor (CAR) T-cell therapy, a revolutionary approach in cancer treatment, reprograms T-cells with engineered synthetic receptors to specifically target and eliminate cancer cells [132]. This approach has shown remarkable clinical success in hematological malignancies, sparking interest in its potential applications for solid tumors, including brain cancer [133]. However, CAR T-cell therapy is associated with a distinct side effect profile, including immune effector cell-associated neurotoxicity syndrome, which can manifest as mild tremors to cerebral edema [134]. Although no direct causal link between CAR T-cell therapy and stroke has been established, several cases of stroke in CAR T-cell recipients have been reported [135,136,137]. The systemic effects of immuno-oncological therapies, such as CAR T-cell therapy, are closely intertwined with cerebrovascular risk in brain cancer patients. Despite offering life-saving benefits, these therapies require careful monitoring and management to minimize the risk of cerebrovascular complications. As the field of immuno-oncology continues to evolve, a deeper understanding of the underlying molecular mechanisms will be essential to developing safer and more effective treatment strategies.

## 3. Therapeutic Interventions of the Neurotoxic Microenvironment

The relationship between brain cancer and stroke is increasingly being elucidated through the concept of the NTME, which is characterized by oxidative stress, neuroinflammation, and BBB dysfunction. This research highlights opportunities to explore therapeutic interventions targeting the NTME to mitigate stroke risk while emphasizing the potential of genomic and proteomic profiling in identifying biomarkers for more personalized treatment approaches.

### 3.1. Targeting the Neurotoxic Microenvironment

The NTME plays a critical role in amplifying stroke risk in brain cancer patients by fostering conditions that promote and sustain vascular and neuronal injury [44]. Therapeutic strategies focused on modulating this environment could have the potential to lower both the incidence and severity of strokes in these patients. Central to the NTME is oxidative stress, driven by ROS and RNS, which inflict cellular damage and disrupt the BBB [41]. Antioxidants capable of neutralizing these reactive species, such as N-acetylcysteine and edaravone, are under consideration for their potential to alleviate oxidative stress, thereby reducing ischemic damage and reinforcing BBB integrity [42,138]. Concurrently, inflammation within the NTME exacerbates stroke risk through cytokine release and immune cell activation [42]. Anti-inflammatory agents, such as specific cytokine blockers targeting IL-1 or TNF-α, could mitigate neuroinflammation [42]. The development of novel drugs that target pro-inflammatory pathways presents additional therapeutic opportunities. Moreover, maintaining BBB integrity is crucial, as breakdown permits harmful substances to permeate the brain, escalating stroke risk. Therapeutics designed to strengthen the BBB, such as matrix metalloproteinase inhibitors or agents that stabilize endothelial cell junctions, could be critical in stroke prevention [139].

### 3.2. Genomic and Proteomic Profiling for Personalized Medicine

Interventions targeting the NTME can be complemented by advancements in genomic and proteomic profiling [42], which identify biomarkers associated with stroke risk in brain cancer patients. These biomarkers enable personalized medicine approaches, tailoring treatments based on an individual’s specific molecular profile [140]. Thus, they enhance therapeutic efficacy and patient outcomes, highlighting the importance of the NTME as a therapeutic target.

By identifying specific biomarkers associated with increased stroke susceptibility, interventions can be tailored more effectively to individual patient needs [141]. Genomic profiling enables the detection of mutations and genetic variants linked to both cancer progression and stroke risk. For example, mutations in oncogenes like EGFRs or tumor suppressors, such as p53, may correlate with hypercoagulability and vascular dysfunction [142]. Meanwhile, proteomic analysis complements genomic data by revealing aberrant protein expression profiles that reflect the NTME pathological state [42]. With this biomarker data, personalized therapeutic strategies can be developed, targeting the specific molecular pathways implicated in a patient’s NTME. This approach might include using specific inhibitors for overexpressed pathways or tailoring antioxidant therapies based on a patient’s oxidative stress profile. Furthermore, integrating genomic and proteomic data into predictive models can enhance the ability to predict stroke events, allowing for prophylactic interventions [143]. These models could consider a range of factors, including genetic predispositions, protein expression levels, and clinical history, to stratify patients according to their risk and guide treatment decisions. By leveraging these advanced profiling techniques, a more precise and personalized approach to managing stroke risk in brain cancer patients ultimately improves patient outcomes and quality of care [141].

## 4. Conclusions

In conclusion, the pathophysiological interplay between brain cancer and stroke is underpinned by cancer-associated hypercoagulability, inflammatory and immunological mechanisms, direct tumor compression, infection-related processes, and treatment-induced complications. Central to this nexus is the NTME, characterized by oxidative stress, neuroinflammation, and BBB dysfunction. The NTME fosters chronic inflammation and stress, creating a milieu that predisposes to and perpetuates cerebrovascular disease. A dual approach that targets the NTME while leveraging advanced genomic and proteomic profiling holds significant promise for improving stroke management in brain cancer patients [144]. Genomic and proteomic tools can identify biomarkers associated with increased stroke susceptibility, enabling tailored therapeutic interventions [141]. Complementing this, predictive modeling powered by artificial intelligence offers a proactive strategy for addressing stroke risks, potentially transforming clinical outcomes for this complex patient population [145]. Future research should prioritize integrating these innovative approaches into clinical practice through well-designed studies and trials. Exploring targeted therapies to mitigate the pathological link between brain cancer and stroke is imperative for improving survival and functional outcomes. Unraveling the intricate interactions between these conditions will refine diagnostic frameworks, advance personalized treatment strategies, and enhance overall patient care.

## Figures and Tables

**Table 1:** Outcomes of studies investigating stroke in cancer patients.

Author, Year	Study Type	Cohort	Brain Cancer Included	Outcome
Cestari et al., 2004 [53]	Retrospective cohort study	96 IS patients with cancer	Primary and metastatic brain cancer included	Lung (30%), brain (9%), and prostate (9%) were the most common cancers. Embolic strokes were predominant (54%). Treatment did not impact survival.
Kreisl et al., 2008 [54]	Retrospective cohort study	68 IS patients with primary brain cancer	Primary brain cancer included; metastatic disease included	Gliomas (60%), meningiomas (25%), and primary CNS lymphomas (6%) were most common. Stroke etiologies included operative complications (49%) and cardioembolic (12%). Radiation effects contributed to 25% of strokes.
Schwarzbach et al., 2012 [4]	Prospective case-controlled study	140 IS patients with cancer; 140 IS controls	Primary brain cancer excluded; metastatic disease included	Higher prevalence of unidentified strokes and multifocal infarctions in cancer patients compared to controls.
Selvik et al., 2014 [55]	Retrospective cohort study	229 IS patients with cancer; 1227 IS patients without cancer	Primary brain cancer included; metastatic disease included	Cancer prevalence was higher among stroke patients. Patients with prior cancers had a higher rate of cardioembolic strokes. Brain cancer accounted for 2% of cases.
Kneihsl et al., 2016 [49]	Retrospective case-controlled study	73 IS patients with active cancer; 227 IS patients with non-active cancer	Primary brain tumors and cerebral metastases excluded	Active cancer patients were younger, had more severe strokes, higher rates of cryptogenic strokes, multifocal infarctions, and poorer outcomes.
Cutting et al., 2016 [50]	Retrospective cohort study	49 IS patients with cancer	Primary brain tumors and cerebral metastases are excluded	Hypercoagulability was the leading stroke etiology (42.9%). Functional outcomes did not vary by cancer type, stage, or duration since diagnosis.
Yoo et al., 2019 [51]	Retrospective case-controlled study	140 IS patients with metastatic cancer; 105 with active nonmetastatic cancer; 223 with nonactive cancer	Primary brain cancer excluded; metastatic disease included	Metastatic disease was independently associated with higher 6-month mortality.
Pana et al., 2021 [52]	Retrospective cohort study	38,855 IS admissions with cancer; 20,895 non-metastatic and 17,960 metastatic cancers	Primary and metastatic brain cancer included	Cancer patients (including brain cancer) had higher in-hospital mortality, prolonged stays, and lower routine discharge rates.
Otite et al., 2022 [56]	Retrospective cross-sectional study	5,748,358 IS patients from 2007 to 2019 National Inpatient Sample	Primary brain tumors and metastases excluded	12.7% of IS patients had previous/active cancer, with metastatic cancer accounting for 34%. Brain cancer cases (2384) were excluded from analysis.
Kikuno et al., 2021 [57]	Retrospective cohort	41 IS patients with active cancer; 51 with inactive cancer; 575 without cancer	Primary brain tumors included; metastatic disease excluded	Infarctions in multiple vascular territories were linked to active cancer. Inactive cancer cases had more atherosclerotic embolic sources. One primary brain cancer was identified in active cancer cases.

Abbreviations: IS = Ischemic stroke.

## Data Availability

The original contributions presented in this study are included in this article. Further inquiries can be directed to the corresponding author.
